# Institutional Engagement Practices as Barriers to Public Health Capacity in Climate Change Policy Discourse: Lessons from the Canadian Province of Ontario

**DOI:** 10.3390/ijerph17176338

**Published:** 2020-08-31

**Authors:** Luckrezia Awuor, Richard Meldrum, Eric N. Liberda

**Affiliations:** 1 Yeates School of Graduate Studies—Environmental Applied Science and Management, Ryerson University, Toronto, ON M5B 2K3, Canada; 2 Yeates School of Graduate Studies—Environmental Applied Science and Management, School of Occupational and Public Health, Ryerson University, Toronto, ON M5B 2K3, Canada; meldrum@ryerson.ca (R.M.); eric.liberda@ryerson.ca (E.N.L.)

**Keywords:** public health framing, climate change discourse, engagement, deliberation, collaboration

## Abstract

Public health engagement in the communication, discussion, and development of climate change policies is essential for climate change policy decisions and discourse. This study examines how the existing governance approaches impact, enable, or constrain the inclusion, participation, and deliberation of public health stakeholders in the climate change policy discourse. Using the case study of the Canadian Province of Ontario, we conducted semi-structured, key informant interviews of public health (11) and non-public health (13) participants engaged in climate change policies in the province. The study results reveal that engagement and partnerships on climate change policies occurred within and across public health and non-public health organizations in Ontario. These engagements impacted public health’s roles, decisions, mandate, and capacities beyond the climate change discourse; enabled access to funds, expertise, and new stakeholders; built relationships for future engagements; supported knowledge sharing, generation, and creation; and advanced public health interests in political platforms and decision making. However, public health’s participation and deliberation were constrained by a fragmented sectoral approach, a lack of holistic inter-organizational structures and process, political and bureaucratic influences, irregular and unestablished communication channels for public health integration, and identities and culture focused on functions, mandates, biased ideologies, and a lack of clear commitment to engage public health. We conclude by providing practical approaches for integrating public health into climate change discourse and policymaking processes and advancing public health partnerships and collaborative opportunities.

## 1. Introduction

Characterized as a “wicked problem,” climate change is a contemporary phenomenon with unparalleled complexity [1,2,3]. It spans several social, environmental, and political issues; it requires the inclusion of various stakeholders and voices with different ideas about the nature of the problem. Achieving such complex yet interrelated needs of climate change management call for sound governance [4,5,6,7,8]. The concept of governance refers to pragmatic, democratic, and decentralized approaches for legitimizing and targeting complexities of climate change policies by accounting for participative structures and deliberative processes that match the scale and intricacies of the issue and the needs for collective decision-making and power-sharing [5,8,9,10]. Adaptive governance, which calls for the management of environmental issues as complex social-ecological systems (SES), has emerged as a pragmatic framework and practice for climate change decision making, as opposed to centralized approaches [4,5,6,7,8]. However, there is also a growing recognition that public health’s participation in climate change communication, discussion, and decision making can advance the adaptive governance of climate change.

Public health framing offers discursive legitimacy in the climate change discourse [11,12,13,14]. That is, the ability to speak on an issue in a manner that influences an understanding of, or actions on, an issue [9,15]. Empirical evidence shows that a public health framed discourse resonates with the public, can support political will, and steer climate change impact awareness and consensus on actions by providing a broader audience with relatable health risks as opposed to the complicated jargons of climate change (e.g., carbon pricing) and articulating the health-climate change connection in the climate change discussions and communications [11,12,13,14]. Lateral public health supports collaborative and democratic platforms for multi-stakeholder engagement that connects the various decision processes, resources, and structures and builds evidence for communicating and integrating health perspectives in policies [16,17,18]. Such platforms also depoliticize climate change by offering bipartisan solutions through broader stakeholder participation, relationships, and knowledge of mutual benefits that impact stakeholder and political will [16,17,19,20]. The health promotion and disease prevention competencies of public health can address the many health impacts, highlight the health co-benefits of actions, and provide the evidence for mainstreaming and linking the health agenda to climate change decisions [11,18,21,22]. The call for broader participation of public health has been reinforced by various scientists and commissions, including the Lancet Countdown, an international multidisciplinary research collaboration that tracks climate change response and resulting health benefits across the globe [19,23,24,25]. Yet, while governments engage in many climate change policy activities at various levels, these efforts are rarely coordinated with public health or embedded with public health needs [26,27]. Evidence also suggests that public health inclusion, framing, and engagement in the climate change discourse remains a challenge, accompanied by distinct context-specific barriers [26,27,28,29,30,31,32,33].

If public health-led policy is relevant in climate change discourse, risk communication, decision making, and consensus-building: why does the empirical evidence reveal a lack of coordination with and inclusion of the sector in climate change governance, and why do public health stakeholders not dominate the discourse? Answering these questions requires an understanding of factors that influence stakeholder engagements in climate change policies, an examination of the interactions between public health stakeholders and other policymakers in progressing climate change policy decisions and discourse, and an analysis of impacts of such interactions on public health’s capacities.

In this setting, the purpose of this study is to examine contexts of institutional engagement in climate change policy discourse, specifically, how the existing governance approaches impact, enable, or constrain the inclusion, participation, and deliberation of public health stakeholders in the climate change policy discourse. To account for the unique geographical contexts and distinct sociopolitical institutions that mirror context-specific ideas, interests, and institutional arrangements [34,35], we use the case study of the Canadian province of Ontario. The study focuses on public health stakeholder engagement and not on the broader issues of public health integration or mainstreaming into climate change policies. Exploring the contexts of institutional engagements in climate change policies can provide policymakers with lessons for integrating public health across a variety of deliberation and participation platforms. Additionally, to break out of the silo approach to climate change governance, evaluation of governance approaches is necessary to ensure the inclusion of public health voices, evidence, and needs in the climate change discourse. Lessons from Ontario can provide context-specific insight into the impacts, complexities, processes, outcomes, and challenges.

## 2. Conceptualizing Stakeholder Engagement and Climate Change Policies

A stakeholder is any person, group of people, or organization impacted by an issue addressed through a public policy [36]. Public policy represents a government’s action or inaction on an issue [37,38,39]. That is, public policy portrays a claim of the legitimate role of the state and politics in the processes and outcomes of a policy and recognizes the role of government in administrative policies and policy instruments [37,38,39,40]. However, achieving the desired policy outcomes require government(s) to engage within (internally), across a variety of organizations and sectors, and with many stakeholders directly or indirectly impacted by an issue.

Broadly, involvement in government policy processes occur through public communication or public participation [41,42]. Public communication is one-way information transmission either from a government agency to the public (public communication) or from the public to an agency (public consultation) [42]. Public participation signifies the government’s approaches to meaningfully engage different stakeholders its policy decisions [42]. Public participation is also synonymous with collaboration, which relates to a mutually beneficial relationship between two or more parties to achieve a specific goal through structured and shared responsibility, authority, and accountability [42,43,44,45]. Collaboration sometimes takes the form of and is synonymously associated with cooperation, coordination, networking, or partnerships [43,44,45]. While partnerships describe any form of engagement, cooperation (formal but independent problem solving), coordination (mutual problem solving without shared resources or authority), networking (information sharing), and collaboration, all represent specific forms of engagement [44,45,46]. Each level of engagement builds on the other (four-tier inter-organizational domains), with collaboration as the most critical tier [44].

Specific to climate change, decision making involves many stakeholders across multiple scales, sectors, and stages of policymaking [19,23]. Many impacts also transcend public health into other sectors due to the multiple pathways of climate change effects and sectoral actions [19,23,47] Collaboration connects decision makers and stakeholders throughout the various stages of a policy [48]. Thus, collaboration is the ideal form of public health engagement in climate change decisions because it connects multiple stakeholders, builds relationships, and supports working towards mutually beneficial actions [43,49,50,51]. Communication and consultation complement participation by providing information that necessitates collaboration [42,43].

Stakeholder engagement in public policy is more than just an interaction between the government and key stakeholders. Rather, the quality of those interactions is defined by the institutional arrangements—structures, responsibilities, processes, networks, and organizations—that impact the integration of a stakeholder in the decision processes [9,38,52,53]. For instance, the failure and lack of inclusion of relevant actors in climate change governance have been blamed on legitimacy, conceived as an outcome resulting from an institution’s activity [9,54]. This is because institutions (socially constructed norms, rules, and principles) supply individuals with reference points—physical arrangements, rules of behavior, values (interests, ideologies, and beliefs), and knowledge—that can influence how, why, when, and where public health is included in climate change governance [9,38,52,53]. In turn, designing and assessing approaches for public health stakeholder inclusion also requires the examination of institutions embedded in the participation structures and deliberative processes [9].

Several researchers have conceptualized approaches and principles of stakeholder engagement in various policies, including public health, environmental, or climate change policy [9,23,36,55,56,57,58,59,60,61,62,63,64,65,66,67]. The literature provides both unique and common factors. However, a framework that integrates these diverse indicators is Cadman’s “theoretical model for evaluating contemporary global governance” [9]. The framework provides two criteria for achieving meaningful participation (interest representation and organizational responsibility) and supporting five indicators (inclusiveness, equality, resources, accountability, and transparency). It also provides two criteria for attaining productive deliberation (decision making and implementation) and six supporting indicators (democracy, agreement, dispute settlement, behavioral change, problem solving, and durability). However, despite such extensive research on institutional engagement approaches, a holistic framework outlining how such factors are weighted and structured in the engagement processes are lacking [57]; but the studies provide essential elements for assessing the levels, impacts, and contexts of institutional engagements.

## 3. The Context of Ontario’s Climate Change Policy Discourse and Public Health Engagement

Ontario is 1 of the 10 provinces of Canada, located in East-Central Canada. Ontario has led, and aggressively engaged in, climate change policies since 2007, mainly under the leadership of the Ministry of Environment, Conservation and Parks (MECP) [68]. The effort began with the signing of the 2007 memorandum of understanding (MOU) between Ontario and California with a collaborative goal of reducing greenhouse gases (GHGs) and promoting energy efficiency [68]. To date, the province has developed numerous strategic action plans and the supporting legislation; held membership in international agreements and initiatives; signed additional MOUs; engaged stakeholders in climate change policy discussions; and developed expert committees [68]. Political and partisan change—from a 15-year government of the Ontario Liberal Party to Progressive Conservative Party of Ontario after the June 2018 elections—have also led to drastic policy changes. These included the elimination of cap and trade program and supporting legislation [69,70,71] and the Environmental Commissioner’s office [72]; reduction in funding or cancellation of climate change programs, green energy funds, and promotion of non-renewable energy consumption [73,74]; repealing the act for advancing green energy [75,76]; reduced focus on climate change in the strategic plan and elimination of the word ‘climate change’ from the ministry of environment (replaced with ‘conservation and parks’); and challenging the federal government carbon tax [77,78,79,80].

In Canada, provinces have autonomy over public health [37]. In Ontario, the public health system is divided into two: provincial and municipal. At the provincial level, the Ministry of Health and Long-term Care (MOHLTC) designs legislation, standards, and protocols guiding public health practice, coordinates emergency response, and accountability [81,82]. At the municipal level, geographically-specific Public Health Units (PHUs) deliver provincially mandated programs but also develop additional policies and programs based on local needs [83,84,85]. Program funding is shared between the two levels of government [86].

To meet public health needs in climate change, the province’s Expert Panel on Climate Change Adaptation (2009) recommended the province assess and expand public health capacity, tools, and resources to enhance public health emergency response to climate change risks, and to complete an assessment of vulnerability and adaptive capabilities within the province’s health regions [86]. Assessment of vulnerabilities and adaptation capacities within the PHUs have been ongoing or completed by other PHUs. In 2013, the MOHLTC’s ‘Make No Little Plan’ laid out the vision, mission, strategic goals, and the public health focus areas. It provided a foundation for the Environmental Health Climate Change Framework for Action to support public health preparedness, evidence generation, and adaptive capacity [81,87]. To assist the PHU in implementing Environmental Health Climate Change Framework for Action, the MOHLTC developed the Ontario Climate Change and Health Toolkits, which includes two complementary tools for the vulnerability and adaptation assessment and a study that models specific climate change impacts [88]. Prior to 2018, the Health Hazard Prevention and Management Standard and the Identification, Investigation, and Management of Health Hazard Protocol guided public health activities on climate change [89,90]. The sector also developed systems for monitoring air quality and extreme heat events. However, it was until 2018 that public health gained an official mandate in the province’s climate change policies supported by the modernization of the Ontario Public Health Standards and the development of Healthy Environments and Climate Change Guideline [91,92].

Within Ontario’s extensive, changing, and challenging climate change policy discourse, public health stakeholders may have interacted with or significantly influenced the discourse and decisions in Ontario. Literature has revealed a fragmented approach and public health constraints in Ontario’s (and Canada’s) climate change governance [26,27,93]. However, to our knowledge, the factors influencing public health’s involvement, how public health has shaped Ontario’s climate change policy discourse, and the impact of Ontario’s institutions on public health’s capacity to participate and deliberate on the province’s climate policy decisions and discourse have never been examined. In addressing these gaps, we attempt to answer the following question:

How have the existing institutions and governance (both processes and structures) in Ontario impacted, enabled, or constrained the inclusion, participation, deliberation, and capacity of public health stakeholders in the province’s climate change policy discourse?

## 4. Methods

This study is part of interpretive and mainstream policy research [94,95,96] that uses the case study of the climate change policy discourse of the Province of Ontario (Canada) to examine the influence of public health inclusion, engagement, and integration in the climate change policy discourse. The study uses semi-structured interviews with 11 public health and 13 non-public health professionals engaged in the decision making of climate change policies and interventions in Ontario.

### 4.1. Sampling and Recruitment

**Figure 1:**
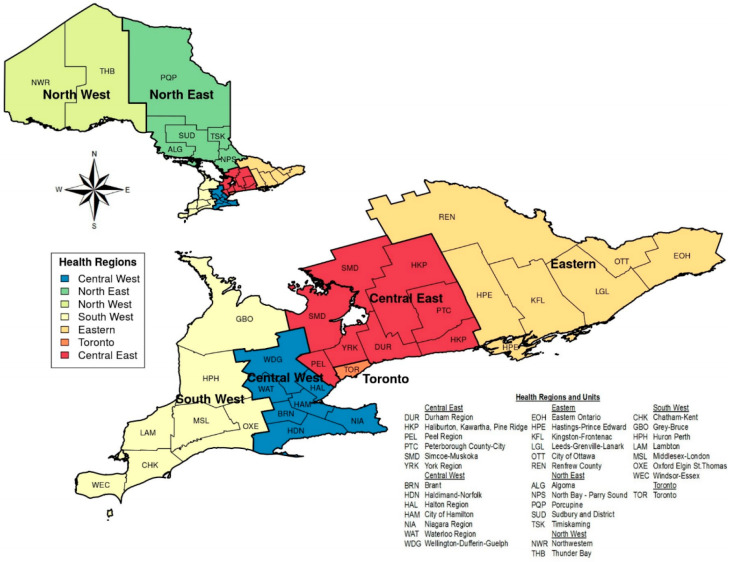
Public health regions and the corresponding public health units in Ontario; Source: Map created using Public Health Ontario boundary files [97].

Cases included the Office of the Premier and 10 provincial ministries, as shown in Table 1, and 7 public health regions (representing 34 local public health units (PHUs)) in Ontario, as illustrated in Figure 1. The cases selected directly engage in the formulation, discussion, and communication of climate change-related decisions at provincial or local levels.

Quota purposeful sampling was used to discriminately identify policy experts with unique or relevant perspective about the study topic by setting a minimum number of at least one participant per case [98,99,100]. Provincial agency participants were identified from the Ontario government website, INFO-GO (a directory of government employees) [101], environmental registry [102] and from grey literature publications by the various ministries. PHU participants were solicited through a research participation request sent to each PHU through the Association of Supervisors of Public Health Inspectors of Ontario (ASPHIO), which comprises of directors and managers serving the 34 PHUs in Ontario [103]. Additionally, we employed snowball (referral) sampling to complement purposeful sampling via all recommendations of qualified experts [100].

### 4.2. Inclusion Criteria

To be included in the study, the participants were required to communicate their experiences, opinions, and events effectively and concisely. Categories such as job positions and roles served as the starting point of participant selection. Participants were current employees or former employees who had had actively taken part in climate change policies within Ontario as well as participated in inter-agency collaborations and interactions with other agencies on matters relating to climate change. They needed to be a public health or policy experts holding leading roles in climate-related areas in respective jurisdictions. The level of responsibilities included managerial, directorship, or persons in senior-level positions, including policy analysts, Medical Officers of Health, environmental specialists, or those holding managerial roles over climate change programs, strategies, policy analysis, planning, formulation, implementation or research. The participants were required to be familiar with the legislation, standards, and policies relevant to climate change actions. A participant who either changed jobs, job description, or position but had previously participated in the climate change policy formulation or implementation at a senior position was considered. Those who did not fall within the sampled jurisdictions, employee type, or without the nature or level of responsibility defined above were excluded from the study.

### 4.3. Data Collection

Telephone interviews were conducted between May to December 2018, using semi-structured, open-ended questions. The interview questions and guiding themes were organized and guided by research themes, as presented in Appendix A and Appendix B. The interviews lasted approximately one hour. All interviews were audio-recorded and transcribed verbatim. The transcripts were then sent to the interviewees for confirmation of content and identification of possible personal identifiers that may have been missed during transcript editing. Data collection (interviewing) was stopped once no new information emerged from the subsequent interviews (theoretical saturation) [104,105,106].

Research ethics approval was obtained from Ryerson University’s Research and Ethics Board (File number: REB 2017-390) and from one of the participating cases, as requested by the organization (File Number: 2018-14). Participation consent was received from all the study participants.

### 4.4. Data Analysis

Interpretive policy research characteristically focuses on meaning construction and symbolic language—written or spoken [95,107,108,109]. On the other hand, mainstream policy studies explore policymaking and agenda-setting processes, policy networks, and governance [95]. Therefore, we use three forms of data analysis. First, focusing on language and discourse, we applied Fairclough’s critical discourse analysis (CDA) to identify texts and themes that describe discourses and engagement practices (including actions, events, and interactions) [34,35,110,111]. Second, we supplement CDA with institutional analysis and analysis of narratives to connect the discursive practices to political contexts of climate change policies, account for social contexts influencing discourse and public health engagement, and to overcome CDA shortfalls. Analysis of a priori and emerging themes was conducted by two coders, focusing on each sentence, and guided by the literature on principles of stakeholder engagement, interview guiding themes, and the CDA checklist (Appendix C). The institutional analysis was guided by the Lomas Model of Decision making framework [112]. We applied the sociocultural approach to narrative analysis by locating the participants’ stories within the sociopolitical contexts of climate change decision making in Ontario [34]. The analysis focused on five areas, as highlighted by Walter [34]. The act done, when and where the act was done (scene), who did the act and how they/he/she did it (agent and agency), and why the act was done (purpose). Lastly, we apply thematic analysis for systematic identification, analysis, and reporting of themes emerging from the data [34,113].

The data management, coding, and analysis were aided by the QSR international’s qualitative analysis software, NVIVO (Version 12). Standardized coding and inter-rater reliability (using Kappa Coefficient) were applied for quality analysis and control [114,115]. The emerging themes explaining the contexts and impacts of collaborative engagements are reported under the two groups (public health (PH) versus non-public health (NPH).

To comply with the ethics requirements and minimize the reader’s ability to connect the various quotes to a specific participant, no unique identifiers are included in participant’s narratives. Instead, the quoted data is assigned as PH or NPH participants. This was required due to the specific focus of some interviewee positions, the personal knowledge that some policy actors may have about the participants, and the political nature of the information provided especially given the political climate at the time of the interviews.

## 5. Results

The results report on the characteristics of participants that produced the study data and the themes that emerged and reported under two headings: influences on public health stakeholder engagements, and effects of interorganizational engagement of intersectoral engagements on public health.

### 5.1. Participant Characteristics

The study data were drawn from 11 PH participants (at least one participant representing MOHLTC and the seven PHU regions) and 13 NPH participants (at least one participant representing the Office of the Premier and 9 NPH ministries), as shown in Table 2. Of the 13 NPH participants interviewed, 5 had previously worked for and engaged in climate change policy decisions with more than one ministry (4 worked for 2 ministries; 1 worked with 3 ministries). Interview questions explored the processes and experiences from those previous ministries as well. Within the PH participants, 2 reported climate change collaborative work with several other PHUs that were not interviewed. Many PH participants were also affiliated with various public health non-governmental organizations (NGOs) in Ontario and worked together in multiple committees or workgroups to promote climate change policy and advocacy. Interview questions explored such engagements. Participants from both groups held positions such as managers, policy experts, policy advisors, policy evaluators, directors, team leaders, and policy analysts. For NPH participants, nine participants have had direct roles in agenda-setting and policy formulation, six participants reported functions in both formulation and implementation, and three participants reported roles in evaluation. PH participants had roles in all stages of policymaking at varying degrees.

### 5.2. Influences on Public Health Stakeholder Engagements

Participation, deliberation, and partnerships on climate change policies occurred within and across public health and non-public health institutions in Ontario. Themes that emerged as influences of public health inclusion, participation, and deliberations in the climate change policy discourse are summarized in Table 3 and are discussed in the five proceeding subheadings.

#### 5.2.1. Fragmented Discursive and Communicative Interactions with Irregular and Unestablished Communication Channels

Interactions were divergent between the NPH and PH groups. However, participants from both groups, (7 PH and 5 NPH participants), acknowledged the ‘silo’ approach to climate change governance in Ontario. The silo approach was evident in the type of stakeholders each group reported to have included in the deliberation of climate change policies, as illustrated in Table 4.

The NPH group portrayed a horizontal approach to climate change engagement (i.e., engagement with stakeholders within similar areas or sectors), a minimal to lack of inclusion of public health representatives (MOHLTC, PHUs, or other health agencies), and extensive engagement with corporations. No direct collaborations were reported between the agencies and the Premier’s office. However, NPH participants (*n* = 8) indicated that the office was represented by the cabinet ministers and their assistants based on their roles within the specific ministries and committees.

Conversely, the PH group reported working with those within and outside the area (or sector) of public health. Engagements with ministries (mandated and non-mandated) were limited, and the PH participants (*n* = 9) reported that there is still a significant gap in public health’s participation in the provincial mitigation and adaptation strategies. Engagements between PHUs and the MOHLTC were mainly related to the modernization of the public health standards, the vulnerability and adaptation assessment needs, and harmonized heat alert guidelines. However, fragmentation was also reported within public health, where some participants reported that larger PHUs within the Greater Toronto Area work closely together without reaching out to smaller PHUs. The four specific PHUs named included Toronto, York Region, Peel Region, and Halton Region. Some participants (*n* = 3) in the PH group also reported experiencing isolation and inability to connect with professionals who could help them meet their climate change policy goals (specifically relating to the completion of the vulnerability and adaptation assessment). A review of collaboration links revealed that such participants hardly reported collaborating with other health units or ministries in their climate change work.

Several NPH (*n* = 8) blamed the fragmented engagement on the status quo (“this is how we have always done it”). Participants also linked the piecemeal engagement approaches to historical relationships, organizational structures or cultures that limited broader stakeholder engagements and partnerships, and political and leadership influences, mandates, framing of climate change. As summarized in Appendix D, some NPH participants believed it was the role of public health agencies to reach out to other ministries for collaboration. Others suggested that the agency’s area of focus did not have a public health perspective or alignment with public health needs. Still, others did not see the role of public health in their policies. Alternatively, when a public health issue is identified, the NPH participants reported that such issues would instead be transferred to public health instead of collaborations.

Many members of the PH group did not understand why public health connections are not made. However, the PH group blamed the lack of elaborate engagement of public health on institutional structures and arrangements that have alienated public health and historically denied public health the legal mandate on climate change policies (*n* = 9). The lack of public health engagement was also associated with the fact climate change was a new area of public health practice. Conversely, a PH participant’s responses also alluded to the lack of relevance of NPH actors, and the relevance of several public health affiliated NGOs, in public health policies, as this quote demonstrates.

*“For us, the sector is the public health sector. We also worked very closely with our key stakeholders. So, our key stakeholders would be the public health units; some of our key public health organizations like CIPHI* (Canadian Institute of Public Health Inspectors), *APHEO* (Association of Public Health Epidemiologists of Ontario), *ASPHIO. We also work very closely with OPHA* (Ontario Public Health Association), *and members from the OPHA.”*

(PH participant)

Progressing the fragmented approach was the lack of communication between the two groups. Although frequent and extensive communication was reported within each group (especially within NPH group), these communications were fragmented, minimal, and irregular between the groups. The lack of interaction between the two groups was obvious in participants narratives through sentences such as: “they are not the one that we have, kind of, the most regular contact with”; “I have never really worked directly with them”; “no, it’s not something that typically comes up”; “no, I don’t remember ever working with the ministries on our projects”; and, “perhaps the ministry (MOHLTC) has worked with them (NPH ministries), but we haven’t on our side.”

Many participants from both groups emphasized the need for better communication to minimize the fragmented approach in Ontario. To leverage communication and engagements between the two groups, the participants (*n* = 18) made several suggestions, as summarized in Table 5.

#### 5.2.2. Influence of Sociopolitical Characteristics of Climate Change Governance

The structures for identifying stakeholders varied between the two groups. However, only PH group (*n* = 8) provided detailed strategic frameworks for stakeholder identification; these included utilization of laid out protocols, guidelines, and workgroups, whose role includes identifying stakeholders for engagement. Historical collaborative contexts were also evident within the PH group, where most participants (*n* = 7) referenced prior collaborations, as ways of identifying stakeholders

As reported in Appendix E, the NPH actors mainly referenced bureaucratic structures (*n* = 9), the use of the Environmental Registry (*n* = 11) and using institutionalized stakeholders (*n* = 5). NPH actors also reported identifying stakeholders based on historical contexts such as previous collaborative projects or deliberations (*n* = 8) and political directions (*n* = 6). Political interests were reported, by the NPH group (*n* = 9), to influence how and who participated in the province’s climate change policies. Political influences were perpetrated by political leaders and senior bureaucrats (e.g., premiers, ministers and assistant ministers), reflected in goals set by the Office of the Premier, and influenced by election promises, strategic framing that are dominated by economic narratives, directives to incoming ministers or through strategic plans or legislation (e.g., a new climate change plan or green energy legislation). As this participant explains, even when public health’s role is acknowledged, the influence of politics determines who takes part in the policy processes.

*“I know that public health can play a role in climate change policies. But as much as I recognize that, the work that we do, and the people that we collaborate with are sometimes determined by political structures, to some degree. We really don’t have an influence on who we collaborate with in climate change policies. Our mandate, for example, and these are mandates that generally come from the Premier’s office, specifies that we work with certain ministries and sectors and so we generally make a point to either directly or indirectly connect with such organizations. So, again, it is politics. Yes, I may have an area where I would like to work with public health in our policies, but our resources and actions are tied to certain actors and not necessarily to public health. Politics basically dictate the direction.”*

(NPH participant)

Participants also reported a lack of direction and leadership from political leaders (Premiers and cabinet ministers and their assistants) and two lead ministries (MECP and MOHLTC) in advancing public health roles or providing supporting structures for public health inclusion. The MECP was reported to play a key role in climate change initiatives and was referred to as the lead agency on climate change issues. All NPH participants reported connecting and deliberating with the MECP. The nature of these engagements were mostly formal, arising from the legal obligations and mandates to consult between the ministries on the various climate change initiatives. However, NPH participants (*n* = 5) suggested that it was the role of MOHLTC or MECP to integrate public health stakeholder engagement needs or the Premier through the mandate letters that direct ministries’ actions, as demonstrated below.

*“Those we work with are mainly determined by the MOECC* (now MECP). *So far, I have not received an indication. I think if you believe that public health needs to be included in* (redacted) *then maybe that has to come from the ministry* (MECP) *or even the mandate letters that give directions to the top bureaucrats.”*

(NPH participant)

Within the public health sector, the MOHLTC was regarded as the lead agency for advancing or steering broader public health stakeholder engagement at the provincial level. Participants reported that engagements and capacities across and between PHUs, and with other non-PHU stakeholders in general, would have been advanced by the MOHLTC. Unfortunately, as this participant highlights, many PH participants (*n* = 9) faulted the MOHLTC for not taking the leadership role or steering the public health sector’s inclusion in climate change policy deliberations.

*“…back to the Ministry of Health… you weren’t finding someone there who is being the champion and leading the way and inspiring others, so this makes it hard.”*

(PH participant)

Even though some NPH participants mention including the ministry of health when public health issues arise, only one concrete example was provided across 13 participants. This related to the inclusion of public health stakeholders in an advisory committee for a disease outbreak to support the outbreak management. However, another example portrayed a transfer of responsibilities to public health for a policy modification instead of working together on the issue to gain consensus or provided hypothetical scenarios of public health inclusion.

#### 5.2.3. Restrictive Structures and Processes of Stakeholder Engagement

Partnerships in climate change policies mainly occurred between actors viewed to have a mandate on climate change or those with mutual interests. This was mainly evident in NPH participants’ narratives. Specific to NPH organizations, these mandates were associated with specific ministries with shared interests. While public health’s mandate in climate change was supported in 2018, interviews with the NPH participants did not reveal evidence suggesting the new public health mandate in climate change had significantly influenced public health inclusion in provincial strategic policy discussions. Many NPH participants (*n* = 8) were not aware of this mandate, and public health was majorly regarded as not having a mandate in the climate change decision- or policy-making processes. In turn, the lack of public health partnerships with ministries was seen as a result of a lack of public health’s role or mandate in the policies that were being formulated or debated. In some instances, NPH participants viewed the provincial policies as superior to those at the local level (e.g., local public health agencies or municipalities). Those at the local level were, therefore, to use such policies as templates for designing their own policies.

To support stakeholder participation, at provincial and local levels, strategic structures included committees, internal collaborative workgroups, professional workgroups, and the Environmental Registry. At the provincial level, engagements heavily relied on government-supported committees (as reported by NPH = 8) that comprised of the various ministries mandated with climate change, with no representation from public health. Such committees facilitated deliberative and participative approaches to climate change decision making resulting in collaborations between the ministries, shaped climate change policies, and influenced consideration of relevant stakeholders. The NPH participants (*n* = 4) also reported that the committees were monopolized by politicians and bureaucrats. However, the committees were also reported to influence relationships between stakeholders. The absence and lack of representation of public health stakeholders in the committees, including the exclusion of the MOHLTC, was acknowledged by PH participants (*n* = 5).

In addition to committee settings, informal communication (i.e., consulting with colleagues) across ministries was also reported by NPH participants (*n* = 6). However, 11 of the 13 NPH participants reported that engagement between ministries and other stakeholders are mainly through Environmental Registry online posts where stakeholder can comment on a government’s policy, decision, or statement of the government on an issue. There is neither communication with the commenters nor individually provided feedback. As one participant puts it, the Environmental Registry is “a government’s way of consulting and engaging on policy issues”. Although the analysis revealed that public health professionals (*n* = 8) had utilized the Environmental Registry to comment on climate change decisions, meaningful consultation between the ministries and public health do not occur, as quoted below.

*“Yeah, so whenever a policy is proposed, we are required to post it in the registry for public comment. We don’t necessarily have a roundtable discussion with many stakeholders unless they are among the ministries that we work with, especially on the various committees we have. But to say that we do have a face-to-face consultation with the public, including public health people, no! But we always receive a lot of comments from many other organizations, including public health.”*

(NPH participant)

Within public health, participation structures for engaging in climate change decisions were also evident. These included structures for engagements within the PHUs; between PHUs and MOHLTC; between PHUs and other municipal departments; and, between PHUs and numerous NGOs. Committees were the predominant form of multi-stakeholder engagement in public health. Some types of committees reported included advisory, steering, workgroup, and technical committees. For instance, steering committees formed to lead and guide the development of public health climate change policy guidelines. Internal committees within PHUs addressed vulnerability and adaptation assessment processes. A technical committee was created to integrate public health mandate in climate change. Committees within municipalities were, in some instances, formed to integrate public health’s role in municipal climate change plans and to facilitate PHUs work with other departments within the local governments, such as planning, water, transportation, and emergency management departments. Within such committees, the main role of public health, as cited by the PH group, was around advancing public health needs in active and sustainable transportation systems. However, a few members of the group (*n* = 3) raised concerns about the gender composition of municipal committees. As this participant notes, the gender imbalance compositions resulted in anxiety and a feeling of inferiority.

*“Some of the meetings I have attended barely have women in them. I think…maybe…this could be just the nature of issues being addressed because I find that many built environment areas are dealing with energy, transportation, or other infrastructure that are dominated by male, especially engineers. But it really makes you so nervous and anxious when I’m putting my points across. Are they even going to consider what I say, or I am even relevant here?”*

(PH participant)

A review of the composition of study participants identified disparities in the gender-balanced composition which were outside of the study’s control. To illustrate, the PH participants that that met the criteria for inclusion in the study, and that were retained or interviewed, consisted mostly of females (82%). Within the NPH group, female participants comprised 32%.

Other than committees, the public health sector’s engagement in climate change policies was supported by workgroups. The two most cited workgroups within public health settings, as mentioned by 9 PH participants, were affiliated with the OPHA, an NGO. The Environmental Health Workgroup directly focused on climate change issues and other related issues such as air quality, water quality, and energy. The Built Environments Workgroup focused on issues related to the planning and implementation of various built environment components and their links to health. Other platforms for deliberation utilized by the group included forums, conferences, and webinars facilitated by Public Health Ontario.

The MOHLTC utilized committees, workgroups, and personal sessions to prepare PHUs for the new requirement of conducting vulnerability and adaptation assessment.

#### 5.2.4. Resources

Resource constraints—namely, financial, expertise, technology, and information—were a barrier to engagement, as reported by both groups. Within public health, the impact of financial resources was the most significant, as cited by all PH participants. However, smaller PHUs and PHUs with large geographical areas reported being most impacted. Other resources cited included lack of data, training, expertise, or technology for advancing public health needs. Despite such barriers, the fragmented approach to climate change governance in Ontario may be hindering public health’s access to relevant data. As this participant narrates, some ministries may have relevant data that are never shared or sought.

*“Because our ministry… and our applied research nature, we have been taking climate change into consideration, probably since as early as the 80s. We may have some long-term data set that we might be able to provide more of a service to public health institutions than they realize. Unfortunately, they don’t reach out to us.”*

(NPH participant)

#### 5.2.5. Ideological Biases

Embedded in the various processes, structures, and interactions were the ideologies that trivialize public health’s relevance in climate change decisions. For instance, lack of public health’s role was implied by the NPH group views of public health belonging to the “humanities” or “social ministries“, or “kind of the health side or the community safety” ministries and as “social-oriented” as opposed to the “applied nature of climate change policies” or “natural resources and infrastructure” focuses of climate policies. Public health agencies were also sometimes referred to as “other” agencies, “not part of the government” or organizations “outside of the government,” signifying the structural features that NPH actors draw upon to identify the relevance of actors in climate change decisions and deliberations.

PH participants (*n* = 6) revealed that attitudes that deprecate public health’s role are very prevalent in their work (both at the provincial and local levels), and not isolated to climate change competencies. In turn, some public health participants reported feeling devalued in various engagement platforms, as illustrated below.

*“That is something we have come across as well. You know, sort of the question of why is public health here?”*

(PH participant)

*“It’s pretty much the same conversation that we have been having related to public health and the built environment. You know, because really the lead ministries related to the built environment are the Municipal Affairs and Housing and Transportation and so through the* (redacted) *we have spent a lot and doing a lot of work on how do we engage the municipal planning sector. And now, how do we engage the transportation sector? And they say the same exact things. Like, you know, why would we invite public health? What interest would they have on a road plan? Same thing, we have talked to transportation engineers, we have talked to transportation planners about if they engage with public health and if so how or where the input would best be and … yeah, the same conversation, different topics.”*

(PH participant)

### 5.3. Effects of Interorganizational Engagements on Public Health

Where institutional engagements were reported—whether across organizations or within the organizations—participants reported four types of impacts: strategic (*n* = 15), resource (*n* = 21), capacity building (*n* = 16) impacts, and opportunities and relationships for future engagements (*n* = 17).

Collaborations within the NPH groups were mainly viewed as efforts to improve strategic roles (i.e., advance the mandate on climate change actions). Resources, including access to skills, funds, and knowledge, were linked to the support for and the outcomes of institutional engagements in climate change frameworks. In turn, NPH participants reported sharing resources to advance the mandated roles through the pooling of funds, transfer of knowledge, and application of technologies relevant to the institutionally designated climate change actions. Within this group, impacts of recent government cutbacks in funds were blamed for the lack of engagement. While the NPH participants reported impacts of collaborative governance on their policies, such narratives mainly related to impacts within the ministries and did not reveal the influences in advancing public health needs in the climate change policy discourse or process.

For the PH group, collaborations expanded their capacities beyond climate change policy needs; supported access to funds and expertise; advanced the sectors approached to policy advocacy, and raising of climate change impacts awareness; created relationships, partnerships, and opportunities for future engagements while also revealing the role of public health in climate change policies; impacted knowledge transfer, sharing, and creation. Strategic impact included strategic planning, advancing public health agenda in various forums, both locally and provincially, including supporting inclusion of mandate, coal powerplants phase-out, and impacting municipal governments’ climate change action plans. Partnerships also supported personal growth by helping collaborators to build confidence and overcome skepticism that such participants felt at the beginning of partnerships; such feelings were overcome as individuals interacted and clearly identified their roles within the broader partnerships. These impacts are summarized in Appendix F.

## 6. Discussion

The analysis of perspectives from the public health and non-public health professionals engaging in climate change policies have revealed how the existing governance approaches impact, enable, or constrain the inclusion, participation, and deliberation of public health stakeholders in the climate change policy discourse. The results reveal incredibly low levels of interaction between the two groups (PH and NPH). Engagements were dominated by networking and cooperation, with collaborations existing within each group. Between the two groups, a few informal relationships existed without commonly defined missions, structures, or goals, and information was only shared as needed. The information-sharing was mainly from public health to the government ministries (public consultation). This cooperation was sometimes seen as a collaboration by public health agencies. On the other hand, some coordination or collaboration existed within each group. Such engagements were defined by both formal and informal structures for accessing information, some resource sharing, and mutual problem-solving. Within each group, evidence was gathered through collaborative networks and communicative platforms.

Fundamentally, discourse brings people together [116,117]. In Ontario, the analysis of discursive strategies revealed four types of discourses guiding Ontario’s climate change policy discourse and propagating public health exclusion. First, authoritative discourse—a closed discursive interaction defined by authority, bureaucracy, and political ideologies—that define the legitimacy of actors in the discursive processes and structures, and produce and reproduce interactions, and reinforce specific procedures and structures of engagement while isolating other stakeholders [116,118,119]. Second, vertical discourse through systematic and standardized processes of deliberations and structures of participation, regulated by mandates and authority on climate change decisions [119,120,121]. Despite efforts to connect between the PHUs, the results reveal that the group was not only excluded from the provincial strategic frameworks, but some were also excluded from each other. Third, the public health sector mainly operated though a horizontal discourse. Defined as a discourse that “entails a set of strategies which are local, segmentally organized, context-specific and dependent” [121]. Horizontal discourse also manifested in functionally focused ministries approach and public health’s (MOHLC and PHUs) independent approaches to climate change governance as dictated by the structure within the ministries and of Ontario’s public health governance that has set them up under geographically specific and independent boards of health. Lastly, the public health sector has applied liberating discourse. This discursive approach liberates the sector from the confines of public health’s formal structures by creating informal spaces for deliberation in climate change policies through affiliation with NGOs [119,122].

The four discourses are propagated by and draw upon Ontario’s institutional arrangements and actors’ values (interests, ideologies, and beliefs) that have shaped the roles of public health in the discourse and provided actors with ideas of relevant stakeholders and knowledge. This is further demonstrated by the large number of NPH participants that declined to participate (see Table 2) because they did not see the role of public health in their policies despite fitting the inclusion criteria of the study. The analysis also reveals Ontario’s functionally specific institutions that have not been designed around public health inclusion–both at the provincial level (MOHLTC exclusion) and local level (PHUs exclusion). These interactions presented five major barriers to public health engagement in the climate change policy discourse. The impediments centred around the lack of holistic inter-organizational processes and structures of engagement, sociopolitical characteristics that influenced collaborative environments, irregular and poorly established communication, inadequate resources, and ideological biases on public health’s roles in the discourse. Where public health stakeholder engagements were successful, those engagements impacted the public health’s ability to support its role beyond climate change decisions, mandate, and capacities in the climate change discourse; enabled access to funds, expertise, and new stakeholders or relationships for future engagements; supported knowledge sharing, generation, and creation; and, advanced public health interests in political platforms and policies (e.g., coal phase-out). The study results correspond with studies conducted in Canada and other developed countries which have revealed a silo approach to climate change governance and the isolation of public health from such structures as well as the lower prioritization of public health needs, and prominence of horizontal collaboration [26,27,123]. Other studies have also identified the influence of discourses and prominence of politics and vested interests on public health’s capacity [30,93,124]. Studies on stakeholder impacts have also linked stakeholder engagement to better evidence-informed policies, effective decision-making, successful uptake and acceptance of interventions, and better communication [36,58,60,125].

Despite strategic and resource impacts on public health stakeholder engagement, the fragmentation of work across climate change stakeholders, inadequate support and sometimes trivialization of public health’s role, and the scarcity of interaction between the public health and non-public health practitioners is particularly worrying. The barriers are not only likely to negate public health capacities in climate change, but they are also likely to minimize climate change impact awareness and GHG reduction requirements in several ways.

Public health organizations have goals to achieve in fighting climate change. They want to minimize the health impacts of climate change, reduce own emissions, and support the development of healthy climate change policies capable of reducing GHG emissions while also protecting populations and advancing coping mechanisms to climate change impacts [19,23]. Achieving these goals requires focusing on a number of factors—social determinants of health, inequity, and injustices—that result from climate change impacts. The goals also rely on primary, secondary, and tertiary prevention strategies [126]. Primary strategies slow, reduce, or stagnate climate change by decreasing GHG emissions. Secondary and tertiary strategies relate to public health adaptation strategies, also referred to as preparedness [126]. Preparedness has a longstanding association with public health practice and is used by health agencies to reduce the population’s vulnerability to the health impacts of climate change. However, mitigation strategies fall outside the public health sector and practice [126]. Institutional engagement is a significant component of building capacities, exchanging knowledge, deliberating on climate change policies, and addressing public health competencies that fall outside of the health sector [112,127]. The lack of inclusion of public health stakeholders is a hindrance to both the management of climate change and public health impacts.

Many climate change health impacts transcend public health into other sectors [19,23,47]. Decision makers may need the help of public health professionals to identify information and processes for integrating health needs into climate change decisions. Additionally, supporting positive health and environmental outcomes and highlighting the co-benefits of action in non-health sectors requires an understanding of the various health components of climate change and social determinants of health [128]. These include an understanding of health impacts at regional and local scales, the vulnerabilities and disparities within the various populations, the risk perceptions and individual risk assessment approaches, and the complex pathways within which climate change and its related actions impact health. While this is true, health impacts may not be entirely understood by other sectors that lack expertise in public health. Rather than applying the sectoral approach to climate change mitigation, public health leadership can enhance the knowledge of the immediate and long-term health benefits of actions to both public health and other sectors, and advance the integration of the benefits of the climate change actions. The knowledge of the mutual benefits can then influence stakeholder and political will [19,20,129]. In effect, the public health sector has an important role to play in shaping the climate change associated health and wellbeing outcomes by influencing sustainable interventions that connect public health to critical climate change debates [126,130]. Through its reports, the Lancet Commission on Climate Change and Public Health primarily advocated for a collaborative approach to climate change by emphasizing the cross-sectoral drivers of health impacts and public health needs [19]. Specifically, the Commission underscores the need for governments to facilitate collaborations between health ministries and other agencies of government in a manner that supports the empowerment of health professionals and integration of health needs in the climate policies [19]. And, as the Commission further advises, the silo approach to addressing climate change health impacts is detrimental to population health in the face of climate change [19].

Advancing public health engagement and participation in the climate change discourse requires holistic inter-organizational processes and structures that recognize the public health roles, mandates, and goals in the discourse. Efforts to include public health in the broader climate change policy discussions and deliberations must also include approaches for breaking out of the silo approach to climate change governance and advancing public health inclusion in climate change discourse. In addition to the suggestions by the study participants (see Table 5), the inclusion of public health in the climate change political agendas are likely to be leveraged in several instances. First, when public health needs are integrated into climate change planning and policy processes (mainstreaming) [18,131,132]. Mainstreaming can support the strategic identification of stakeholders, the definition of mutual goals, collaborative needs, and resources for jointly addressing climate change. Mainstreaming can occur when all sectors of governments systematically consider the social and health consequences of proposed climate change policies (health-in-all policy (HiAP) [18,131,132]. Advancing a HiAP approach to climate change policies can permit the integration of public health voices and evidence and the visualization of social determinants of health, inequities, and injustices of climate change policies. Mainstreaming can also occur when governments consider the human-animal-environmental health interface (One Health) and the resulting need for a multisectoral and multidisciplinary collaborative approach [133,134,135]. The One Health approach requires coordination of climate change policy activities, policy processes and discussions across institutions, sectors, and organizations to minimize the silos and support communication, knowledge exchange and awareness of each other’s goals, mandates, areas of focus, roles and responsibilities that support the achievement of human–animal–environmental health outcomes which are also inextricably linked to climate change [18].

Second, there is a need for a climate change governance approach for multi-stakeholder inclusion. Deliberative (collaborative) governance, is a multi-stakeholder engagement approach, where governing institutions or agencies support participation by collectively working with and integrating values from a variety of stakeholders [41,42,132,136,137,138]. The approach stresses the creation of partnerships where the need for a two-way communication and knowledge exchange is paramount [139]. As a result, the engagement processes initiated by either an agency or public health institution should bring stakeholders together through deliberative forums. An approach to achieving collaborative governance can be through framing for deliberation. Framing portrays a policy image and forms the basis for the deliberation and decision making of policies and advancing [17,140,141]. Advancing a policy image that considers a wide range of options and clarifies the different approaches to addressing the issue provides unique opportunities for broader stakeholder engagement in the political and elite-driven governance processes of climate change policies [11,13,142,143]. Specifically, including a public health frame has been shown to provide an approach for uniting the different stakeholders within the climate change dialogue [11,16,19,29,144]. Equally, collaborative governance implies that meaningful institutional engagements are not merely one-way information provision or consultation but rather a process where both the agencies and the public health organizations are participating and engaged in the decision-making process. Participation is situated as a form of public engagement where diverse stakeholders are included in the process as opposed to the “traditional models of governance’ that only include mandated policymakers and select experts [41,42]. While this collaborative governance approach is necessary, an integrative framework that conceptualizes and legitimizes public health’s discursive legitimacy and meaningful participation in governance processes and structures of climate change policymaking is lacking. The study results and interpretation postulate that advancing the public health frame in the discourse requires the legitimation of public health into the various institutional structures and governance of climate change. Future research can support the need by developing a framework for conceptualizing and guiding public health inclusion in climate change governance.

The paucity of the inclusion of local health agencies (PHUs) in the provincial climate change policy deliberation structures also shows that the province has not meaningfully included local municipalities in climate change policies, which may also imply that municipalities do not fit in the current model of provincial climate governance. The lack of inclusion of local governments in the broader climate change governance structures has been documented [145,146]. Such deficiencies are accompanied by limited public support and resources, competing priorities, inability to provide the local governments with opportunities for policy development, and inadequate climate change discussions, as were also reflected in our study [145,146,147,148].

The findings suggest that engagement in climate change policies is embedded in power influences portrayed in places and spaces of participation and politics and determined by the legitimacy of actors in the deliberation processes. For instance, despite public health’s ability to create some spaces for engagement, their participation is constrained by restrictive deliberation platforms and the lack of equal opportunities for participation. The spaces for participation were defined by mandates, legislative rights, and formal invitation (e.g., to committees), of which public health was not included. Institutions can exercise power in several ways. Power may be visible, invisible, or hidden; it can be legitimate or illegitimate or can be concentrated (elitism) or diffused (pluralism) across institutional settings [38,149]. The influence of power dynamics in limiting actors’ spaces and places for participation and deliberation on a variety of policies has been evidenced in a variety of literature. These have included climate change [146,150], the environment [151], health [152], economic inequality [153], mental health [154], participation and governance [155], policy processes [156], and water management [157]. The first approach to changing or balancing power relations is to understand how power works to influence policy engagement and outcomes [149,158,159]. While the analysis of power was beyond this study, future studies can investigate the influences of power dynamics and the resulting institutional processes, structures, and their implications on public health engagement in climate change. Such knowledge can support resources for integrating and legitimizing public health’s role in the climate change discourse.

Gender disparity emerged as an influence on public health participation in climate change policies at the local level in Ontario. Climate change policies are intended to impact people’s choices and behavior, and each policy has a gender perspective or impacts. There is also a growing recognition and awareness of the influence of gender diversity in advancing meaningful and diverse participation that reflects the needs of the society, better science, and relevant evidence [160,161,162]. As a result, the lack of inclusive and diverse participative and consultative platforms are alarming. Gender mainstreaming is a strategy that has been used to account for the gender sensitivity, diversity, equality, and women’s perspectives in policies and programs proposed and forwarded by institutions [163,164]. The approach can be legitimized through discursive practices that integrate the application of language that influences how women are perceived in consultative platforms or through institutional approaches that integrate the voices and needs of diverse groups in policy areas [163,164,165]. Institutional advances can include strategic approaches such as political commitments and legally binding frameworks (e.g., tools for assessing statistics, budgeting, impacts, stakeholders, etc.) for integrating gender perspectives into policy contents [166]. Such approaches can also include the establishment of institutional frameworks that highlight approaches for integration as well as the roles and responsibilities [166]. Gender-based plus (GBA+) is a framework commonly used, specifically in the Canadian federal government consultation processes, to overcome the gender differences and the historical or power structures of public participation in policies [160]. However, given this study’s limitation on gender integration, future studies can examine the strategic approaches and political commitments to gender inclusion in Ontario’s climate change policies, since gender emergence as in impediment to public health engagement. Such studies can benefit from a survey, using large study samples that also examine additional social characteristics (such as age and education), that allow for would also allow for statistical inference of gender influences and additional social characteristics (e.g., age and education), as opposed to the qualitative, function-specific approach used in this study.

## 7. Conclusions

Over the years, the significance of public health engagement in climate change policy discourse has been advanced and emphasized. Our study suggests that there still exists contextual and systemic institutional barriers to the sector’s involvement in the processes, discussion, and communication of climate change. We have identified five broad elements and the corresponding factors that hinder public health’s effort in climate change policy discourse. Such factors relate to the influences of collaborative environments (how stakeholders are identified, political, and bureaucratic influences and leadership that steer cross-sectoral collaborations), institutional structures and process and characteristics of collaborators as depicted in the goals, responsibilities, mandates, ideologies, interests, and identities or characteristics of collaborators, resource constraints, and the lack of established and supported cross-sectoral communication. Despite the challenges, stakeholder engagements impacted public health’s roles beyond climate change decisions, mandate, and capacities in the climate change discourse; enabled access to funds, expertise, and new stakeholders or relationships for future engagements; supported knowledge sharing, generation, and creation; and, advanced public health interests in political platforms and policies. To meet the public health and adaptive governance needs of climate change, holistic inter-organizational processes and structures that recognize public health needs are necessary. These can be achieved through mainstreaming of public health in the broader climate change policies and through deliberative approaches that legitimize the participation of different actors in the discussion of climate change policies.

## Figures and Tables

**Table 1:** Case study sample from provincial agencies

Office of the Premier (OP)
Ministry of: Agriculture, Food and Rural Affairs (OMAFRA) Health and Long-Term Care (MOHLTC) Municipal Affairs and Housing (MMAH) Natural Resources and Forestry (MNR) Transportation (MOT) Research, Innovation and Science (MRIS) * Economic Development, Job Creation and Trade (MEDJCT) ** Environment and Conservation and Parks (MECP) *** Energy, Northern Development and Mines (MENDM) **** Indigenous Affairs (MIA) *****

In June 2018, changes (none impacting our study) occurred to some of the ministries selected, as reflected below. * Eliminated. ** Previously—Economic Development and Growth. *** Previously—Ministry of Environment and Climate Change. **** Previously—Ministry of Energy. ***** Previously—Indigenous Relations and Reconciliation.

**Table 2:** Participation solicitation, response, and retention

Participation	NPH	PH
Number of participants solicited	84	34
Total response received	71	18
Declined to participate:		
Did not see the role of public health in their work	46	-
Lack of role or mandate on climate change	-	1
Interviewed and retained (includes four referrals from NPH and three from PH groups)	13	11
Accepted to participate but not interviewed (theoretical saturation)	5	6
No reasons provided for declining participation	7	-

**Table 3:** Main themes emerging as influences of public health stakeholder engagement, as guided by CDA, analysis of institutions and narratives, and institutional engagement elements from the literature review

Features	Definition	Emerging Influences on Public Health Stakeholder Inclusion
Fragmented discursive and communicative interactions	Strategies and approaches for facilitating discussions and communication between PH and NPH sector	- Closed discursive interactions defined by authority, bureaucracy, and political interests or directives that determine legitimate actors - Structured, functionally focused, systematic discursive approaches within NPH ministries and public health agencies - Lack of established channels of communication and irregular communication between PH and NPH agencies - Strategic framing of climate change in economic terms that set boundaries of climate change decisions, the image of climate change, and relevant external stakeholders (e.g., corporations as dominant external stakeholders)
Sociopolitical characteristics of climate change governance	Social and political environments or group characteristics that support or negate collaboration with public health agencies	- Lack of frameworks and strategies, within the NPH sector, for identifying, accessing, and integrating public health stakeholders - NPH ministries have a history of collaborating with each other; prior collaborations within each group formed the basis of future collaborations or offered potentials for future collaborations - Stakeholder identification is highly influenced by bureaucracy and political influences or interests. - Paucity of public health inclusion blamed on the lack of leadership from the two lead ministries (MECP and MOHLTC) who have failed to champion the needs of or integrated public health into the climate change governance, and political leaders (premier leading the government in power and their cabinet—ministers and their assistants) who have influenced the agenda, goals, legitimacy of actors, and resources for collaboration
Restrictive structures and processes	Formal institutional structure and processes for participation and deliberation	- Formal rules, legislation, plans, and structures that define roles and authority over climate change policies leading to domination by specific actors, specifically elites (experts, bureaucrats, cabinet, civil servants, and corporate representatives). - Mandates have assigned ownership to climate change decisions and do not account for flexibility in consideration of public health stakeholders; have also led to a difference in goals and approaches of deliberations between public health and non-public health agencies - Decentralized policymaking between provincial and municipal governments and a lack of integration of municipal governments into the current model of provincial climate change governance, has led to the exclusion of local public health stakeholders in deliberation forums - Domination of formal institutions (e.g., cabinet ministers and assistants) and sectors (e.g., energy, transportation, industry) in the discourse that have resulted in horizontal collaborations along functional and sectoral lines without the inclusion of MOHLTC - Rules (legislation, strategic plans, budget, policies, constitution) that provided authority to and legitimize the role of actors while lacking clear roles for public health. - Strategic platforms, such as committees that do not include public health participants (including the MOHLTC which is a provincial ministry unlike local PHUs) and Environmental Registry which do not provide meaningful participation - Geographically distinct decision making within the public health sector without extensive coordination or accounting for resource constraints and geographical size of PHUs - Gender imbalance in municipal policy processes
Ideological biases	Views of whether public health is a relevant stakeholder	- Trivialization of public health’s role in climate change decisions through statements that imply the insignificant role of public health and attitudes that deprecate public health’s role - Inability to compromise on public health inclusion because they are considered not have a mandate in climate change
Resource constraints	Money, expertise, and information for advancing public health’s capacity in the discourse.	- Inadequate funding for advancing public health needs in climate change governance - Lack of adequate expertise, information, and training for advancing public health’s capacities

Some of the emerging themes may appear in more than one element because they apply to both the elements of discursive approaches and institutional influences.

**Table 4:** Summary of stakeholder included in climate policy deliberations

Collaborated with	NPH (*n* = 13)	PH (*n* = 11)
Mandated ministries	13 *	4 **
Non-mandated ministries (excluding MOHLTC)	3	0
MOHLTC	2	6
PHUs	0	9
Federal government	3	8
Municipal government	2	7
Communities	0	4
Non-governmental organizations	3	5
Health agencies (other than PHU)	0	6
Academia	2	4
Corporations	11	0

* Participants reported engaging with at least 4 or more ministries; ** Participants reported engaging with no more than one ministry except one participant (who reported engaging with two ministries).

**Table 5:** How to leverage communication between PH and NPH stakeholders

Presence of framework for learning about each other’s roles, responsibilities, and leadership areas Availability and access to tools for stakeholder identification and integration Development of communication channels that facilitate open, two-way communication between the two groups, unlike the currently used Environmental Registry Integration of needs of public health in the mandates, with clear goals and objectives and clarifications of points of engagement Involvement on each group in respective climate change initiatives from the initial stages of policy development, implementation, and evaluation

## Appendix A. Interview Guide

**Overarching Themes**	**Questions**	**Possible or Additional Probes**
**Policy background**	Can you please tell me a little about your position and the responsibilities you have in this post?	What roles do you play in [insert organization]?
	Please tell me how you have addressed issues about climate change in [insert the sector/organization] policies	When did that happen? Who else was involved? How does your role relate to the broader climate change strategies in Ontario? What are some of the reasons for engaging in such policies? What are some of the accomplishments you have achieved in your engagements?
**Policy processes**	What is the mandate of your organization in climate change? How are climate change decisions made? Are there policies that have/have not worked? What were the reasons for the successes and challenges?	Priorities and focus Policy influences Is public health integrated/relevant in the decision-making structures, and processes? Policy evaluations
**Meaning of climate change**	What does climate change mean to you? Are such definitions shared amongst the actors in the organization?	When you think about climate change what comes to mind and how does that inform the policies that you develop?
**Collaborations**	Have you worked with other institutions/sectors/ministries to support climate change actions?	Who were they? When did this happen? What was your role? How did that come about? How did you feel about your role?
	Are you aware of any climate change action plan/document/policy that your office/organization has been a part of? If none, are there any plans for future policies or actions? **OR** Has your organization collaborated with other ministries/organizations to support climate change actions? **OR** How does your [insert organization] work with other institutions/sectors/ministries on climate change issues?	Would you elaborate on that? When did this happen? Did you have a role that you would like to elaborate on? Could you mention other sectors included in this (these) policy process(es)?
	Given the number of sectors that influence climate change policies, is there ever any overlap or conflicts? If yes, how are disagreements resolved?	What challenges have you experienced given the intersectoral nature of climate change policies?
	Have you tried to include pH professionals into your climate change strategies to minimize such conflicts?	(this question presents an opportunity for asking questions relating to consideration of public health when challenges emerge)
	Have you tried to integrate public into your health climate change policies? If yes, how so? If not, why not?	Has public health played any role in your climate change policy processes?
**Evidence**	What type of evidence do you consider when developing and deliberating on climate change policies?	How are such evidence accessed? How are the needs determined? How is the evidence integrated into climate change policies?
	Are public health evidence considered?	How are such evidence accessed? How are the needs determined? How is the evidence integrated into climate change policies?
	Do you utilize decision support tools?	What types are utilized; how, when, where, and why?
**Perception of public health inclusion** **Public health’s role/leadership**	What is your view of health professionals advocating for the inclusion of public health the climate change frameworks?	Are climate change and public health connected?
	Do you believe that public health has a role in leading role in climate change actions?	
	What is your view of health professionals advocating for a public health frame of climate change?	
	Do you think the inclusion of public health in the core climate change frameworks is essential for sustainable climate change actions? If yes, how so?	Could you tell me more about that?
	In the long-term, do you think public health has a place in the climate change discourse?	If yes, how so? If no, why not?
	Do you think a public health frame is relevant to your organizational policies?	
	Can a public health leadership be emphasized when others (such as the MOECC) are leading climate change discourse?	
	Has anyone ever asked your views on the inclusion of public health on climate change policy processes? If yes, how often?	If yes, what impact did this have on your policies? If not, why do you think public health has not been considered in your policy discussions?
Closing	Is there anything else you would like to add to what we have covered?	
	Can I contact you later in case I have any questions?	

## Appendix B. Interview Questions Guiding Themes

**Policy background** - Position - Responsibilities - Reason for engaging in climate change - Accomplishments and policies addressed
**Climate change policies and processes** - Organizational policy goals, mandate, priorities, focus, and decisions - How policies are developed - Structures for decision making - Principles for developing climate change policies - Influences on the decision processes - Integration of public health in the structures - The relevance of public health in organization’s climate change policy processes - Successes, challenges and opportunities - Overcoming conflicts and overlaps between different jurisdictions - Opinions on realistic climate change policies - Opinions on past and current directions of Ontario’s climate change policies - Opinions on policies that have (not) worked out well - Policy evaluations
**Meanings of climate change and public health needs (personal and professional opinions)** - Definition and understanding of climate change - Climate change framing - Public health framing - Whether meanings of definition and frame of climate change are shared the organization
**Stakeholders and collaborations** - Types of stakeholders - Roles that stakeholders identified played - Types of climate change policies influenced by stakeholders - How stakeholders are identified and consulted - How the organization works with stakeholders - Current structures and processes for stakeholder engagements and participation - Opinions on current structures and processes of stakeholder engagements and participation - Impacts of stakeholder engagements
**Evidence** - How evidence needs are determined - Types of evidence considered - How evidence is accessed - How evidence is integrated into climate change policies - How public health evidence and needs are considered, accessed and integrated - Types of decision support tools used - Evidence associated challenges - How evidence or information is shared - Evidence or information gaps
**Perspectives** - The connection between public health and climate change - Public health’s role in climate change - Public health’s leadership - Public health framed climate change - Ideal roles for public health in climate change - Inclusion of public health in climate change - Whether perceptions are shared by others in the organization or stakeholders worked with

## Appendix C. Main Issues Analyzed in Textual Analysis and Discursive Practices

**Thematic frames** (climate change framing in Ontario) - Involved analyzing the historical presentation (framing) of the climate change issue. - The theme only focused on preexisting documents (from government websites) to establish how climate change has been historically presented in Ontario (between 2007–2018) - A priori themes guiding frame analysis; the themes included economic, environmental, health, moral, and scientific frames. - Analyzed how the frames were ordered and the narratives (rhetoric) that guided the framing.	**Genre** - Represents ways of acting or interacting [e.g., meeting] or style used in the text [e.g., a persuasive or descriptive text] (Fairclough, 2003; Walter, 2013). - Analyzed the types of genres and their characteristics; activities regulating and managing climate change decision processes within institutions (e.g., types of options for participating in climate change decisions). - Analyzed genre mixing (e.g., activities for promoting climate change decisions locally or internationally or alternating reporting structures using a mix of quotations and facts in a single report).
**Intertextuality** - Involves the processes of embedding other texts within a text—that is, integrating other voices into the texts other than the voice of the author (Fairclough, 2003; Walter, 2013). (E.g. references or embedding direct quotations within a document or providing links) - Analyzed texts for links, quotations or any form of embedded in texts other than those of authors.	**Discourse** - The type of discourse drawn. - Analyzing the discourse guiding Ontario’s climate change actions was guided by Dryzek (2013) and included analyzing the types of entities whose existence is recognized or constructed; the presence of assumptions, metaphors and features characterizing climate change discourse (s); characterization of agents and their motives - Identification of orders of discourse; that is the power relation between the writer or speaker and the reader or listener if interviewing (Fairclough, 2003).
**Style (identificational meanings)** - Relates to how people identify themselves and are identified by others within a social setting (Fairclough, 2003). - Analyzed social (e.g., structure and agency) and personal identities and features that characterize the styles drawn upon by actors (including body language).	**Social differences** - The salience of identities (e.g., identifying to belong to a particular group). - Analyzed scenarios that characterize, recognize, accentuate or explore differences; how the differences are claimed (e.g., consensus over or normalization of, attempts to solve or suppression of the differences).
**Social events** - Are practices within institutions that lead to the selection of specific structures and exclusion of others (Fairclough, 2003; Walter, 2013). - Texts were coded for the emergence of themes related to social events and chains of social events (e.g., how the dominant frame is embedded in the networks of structures within institutions). - Whether the text was part of or network of texts.	**Representation of social events** - Analysis of how the social events were presented (e.g., abstract vs. concrete); the elements of social events represented; how the processes of the social events were presented and the predominant process types; metaphors applied; how social actors are represented (e.g., named, generic). - Analysis of presentation of historical events or milestones.
**Assumptions** - Refers to the implicitness of texts and based on three types of assumptions: existential (“assumption about what exists”), propositional (“assumptions about what is or can be or will be the case”) and value (“assumption about what is desirable”) (Fairclough, 2003). - Fairclough (2003) uses globalization as an illustrative example. That it is assumed that globalization exists (existential) and that it is a process (propositional) which can ‘help’ achieve economic progress (value). - Analyzed the various assumptions, perceptions and ideologies present in texts.	**Modality** - Defined by Walter (2013) as a term for signifying “author’s or speaker’s commitment to the claim being made.” - Related to speech functions in that modality supports the analysis of “what people commit themselves to when they make statements, ask questions, make demands or offers” because each signals a different commitment (Fairclough, 2003). - Analyzed text for commitments, attitudes, judgements and stances of actors.
**Actional Meanings (exchanges, speech functions, and grammatical mood)** - Exchange: relates to exchange in dialogue (Fairclough, 2003; Walter, 2013). Analysis of exchanges focused on two types of exchanges: knowledge and activity exchange. In knowledge exchange, analysis focused on the exchange of information, how information is given and elicited and how facts are stated. Activity exchange focused on the analysis of activities that people do or get others to do. - Speech functions: relate to acts of dialogue (Fairclough, 2003; Walter, 2013). That is, is an analysis of whether the knowledge exchanges are initiated by actor or by others through, for example, offers or demands; and whether activity exchanges are initiated by the knower(s) or others through, for example, questions and statements). - Grammatical mood: analyzed how exchanges and speech functions (dialogues) are portrayed in sentences (e.g., declaratory, interrogative or imperative sentences)	**Meaning relations** - Semantic relations are presented in grammatical structures of sentences; that is relations between sentences and clauses (or simple sentences) within sentences in a manner that legitimize the issue or creates classes of differences (e.g., power). - Example by Fairclough (2003). “We are late because train was delayed.” Conveys a causal meaning relation. The underlined word acts as a grammatical connector to elaborate a causal relationship indicating that the reason for the delay is due to train arriving late. - Involved analysis of word connectors which mark relationships and their relationships (e.g., causal, conditional, temporal, additive, elaborative).
Source: [34,111].	

## Appendix D. Contexts of Collaboration

**Attribute**	**No. of Responses**	**Sample Quotes**
Would collaborate only when public health reached out	N = 7	“I think if public health approached us for inclusion in one of our processes for climate change, we would consider such partnerships. Maybe they just need to explain to us a little bit more of how their involvement will benefit or complement what we are currently doing.” [NPH participant]
Would collaborate only on known areas of public health or a public health issue is identified	N = 8	“I believe we would collaborate with public health if what we are working on issues directly touched public health mandates… such as those related to climate change and its impact on diseases. But again, those are not the focus of our policies. So it may be hard to say that, yes, I will include public health.” [NPH participant]
When PH focus is identified, the issue is transferred to PH	N = 9	“If there is a public health implication in this policy proposal, we are going to move it to the public health people. Like I think they have a public health division there.” [NPH participant]
Do not collaborate with PH at all	N = 10	“No, no involvement of public health agencies.” [NPH participant]
Public health is responsible for initiating collaboration	N = 8	“No, I don’t think there is like a specific topic health perspective. It is like that’s really the responsibility of the ministry of health, public health is their mandate,…so it’s up to them to ask other ministries to consider public health.” [NPH participant]
Acknowledge public health relevance but do not collaborate	N = 6	“I would say I’ve been asked what the public health ramifications of some of our findings might be but to say that I’m being inclusive, or including public health, I can’t say that we do that definitively, no.” [NPH participant]
Do not see the role of public health in their work	N = 6	“No, I don’t think there is like a specific … health perspective.” [NPH participant]
		“I have not worked with public health or consulted public health in any of the policies we developed. I don’t think there was any role that public health could have played in our policies.” [NPH participant]
Public health is only a small consideration of the overall policies	N = 6	“We deal with broad climate change issues and when you consider public health issues, it is only a small proportion of what the climate change policies target. I think at this stage in climate change policies, we can’t target every little impact that climate change poses…I mean, I don’t think we are there yet…I think that what current policies and decisions are targeting are more broadly defined.” [NPH participant]

## Appendix E. Structures for Stakeholder Identification (as Reported by the NPH Group)

**How Stakeholders are Identified**	**Sample Quote**
**Bureaucracy**	“More specific issue is the bureaucracy itself. And ministry always has, sort of, known stakeholders. Like people or specific organizations that they have reached to before, that would typically be …. anyone who would write to the minister. Generally, there is a tradition in Ontario that people who contact the Minister might have an opportunity to talk directly to the political level about a topic. So, in theory, there are people and organizations who have the opportunity to meet with the politicians to talk about climate change, for example. And then there are also organizations with whom the administration might need informally to understand more about what actors are working on out there, and how the government policy design might leverage and work with what’s already happening. For example, on climate change information, we were very aware of not getting in the way of organization that makes it their business to translate climate change data, and instead, designing a government program that would build on the activities already taking place in municipalities and private sector.” [NPH participant]
**Environmental registry**	“In terms of structure to identify stakeholders in Ontario, it really is the Environmental Registry that is used to identify number of stakeholders and to understand the public need and the challenges and gaps that might be important when designing a new environmental policy.” [NPH participant]
**Internalized stakeholders**	“I mean some of them (stakeholders) just become internalized, and people go on” [NPH participant]
	“At the end of the day, every ministry has their share of stakeholders who they put at the forefront.” [NPH participant]
	“We have those stakeholders that we have worked with on other climate change decision processes, and we turn to them when similar platforms arise.” [NPH participant]
**Political direction**	“They [the government] would give the political level some direction, always.” [NPH participant]
**Approach the MOHLTC**	“I think if we were looking for specific public health stakeholders or health stakeholders, we would go to the Ministry of Health for their thoughts.” [NPH participant]
**Historical contexts**	“The ministry has demonstrated long-term collaborative work with partners across ministries who engage in climate change. These collaborations did not begin with climate change, but we have also engaged in other projects that impact the environment in general. And so whenever something new comes up, like climate change, for example, we have these people, from ministries, that we are already working with and who we know their expertise... I think it makes it easier that way, rather than finding new partners to work with.” [NPH participant]

## Appendix F. Impacts of Engagements on Public Health’s Capacities

**Impact**	**Sample Quotes**	**Level of Influence**
**Knowledge exchange**	“[collaboration] provided us with a forum to do a lot of knowledge translation and a lot of knowledge exchange, and …. I have worked with other health units that are now in the process of doing it [V and A assessment]. So I have worked with [redacted], I have worked closely with [redacted]. Each community has to do a vulnerability assessment in their own way, but there are lessons that can be learned, and so I have met with them. I have had discussions with a number of other health units, [redacted] in [redacted]. Just this past week, I have had some discussions with [redacted] in [redacted]. So they are looking at doing vulnerability assessment similar to what we did and so, you know, lessons learned, those kinds of thing.” [PH participant]	Local
**Learning, knowledge sharing and exchange, and skills development**	“It was an enormous learning curve for me, but it was really interesting because I brought perspectives for it that perhaps we wouldn’t have thought of, and it was really interesting dialogue, and we all collectively learned a lot from each other.” [PH participant]	Local
	“I am learning from my colleagues as much as they are learning from me, and so you know, you take the lessons learned back, and you incorporate it into the work that you are doing. So it is a lot of capacity building; it’s a lot of knowledge translation, those kinds of things.” [PH participant]	
**Access to resources**	“One piece that we identified within our key informant interviews was the need for a regional network that could help to bring different stakeholders together to support climate change mitigation and adaptation, and that’s because most of our municipalities are quite small and they are resource-strapped, and they don’t have either the money or the knowledge to be able to start some of the climate change adaptations. And so they wanted a way that they could have a community of practice or a sharing network or a way that they could collaborate to create regional actions. So the health unit has been working to pull that group together, and it is something that we are going to be continuing to lead over the next couple of years. We are calling it a [redacted], where we have conservation authorities, school boards, municipalities, and ourselves as health unit partners working together to identify what others are doing, where there could be collaborations and partnerships and what funding opportunities could be done collaboratively. And so it was one of those pieces that everyone sort of said we need this, but no one was able or had the resources to step up and lead. And so, we were able to put those resources into that program, and we will continue to do so.” [PH participant]	Local
**Building capacities beyond climate change needs**	“So I think it is an opportunity [collaborating with others] that other health units hopefully can take up as well, and we have done it for other program areas, for social determinants of health, for healthy built environment. Public health has been engaged in community development and engagement for many years, and so I think this is just another area that public health can lead in.” [PH participant]	Local
**Advocacy**	“As well, I sat on the vulnerability assessments from [redacted], so as I mentioned—water, wastewater, infrastructure and so on—and brought forth a public health lens to those vulnerability assessments.” [PH participant]	Local
**Strategic**	“That work [that is, a vulnerability assessment that the participant did in collaboration with other stakeholders] has driven much of the work that we have done since”. [PH participant]	Local
**Platforms for potential and future partnerships**	“What that [a previous collaboration] also did was set stage for further partnership work, and we have done a couple of interesting projects with partners that perhaps we wouldn’t have worked with as closely in the past. For example, we developed a tool to look at [redacted] and brought in a social-economic lens, brought in a health protection lens. Looking at things like urban heat island. Looking at equity, all those kind of issues, … we are now doing a pilot project applying that tool in [redacted] and looking at communities that are disadvantaged for one reason or another and looking to see if we can try and impact things like the urban heat island. So there has been a lot of involvement in a lot of different projects and people have been, in my mind, very welcoming of the public health lens to the discussion. The engineers welcomed us; the planners have welcomed us. There has been learning of each other’s languages and needs, and that takes time, but certainly, there has been no hesitation, whatsoever, to have public health sitting at the table.” [PH participant]	Local
**Inclusion of public health mandate**	“[redacted] that promoted or pushed to have more information or more requirements in the public health standards related to public health action on climate change, and I do know that a lot of the health units are doing that work.” [PH participant]	Provincial and local
**Accessing hard-to-reach communities**	“We did try to engage with our First Nation and Metis communities within the region. We were unsuccessful in engaging in that for the vulnerability assessment key informant process. I think we just didn’t have the right contact. We weren’t connecting with the right people. I personally didn’t have any connections with any of our first nations partners from previous work, and like, I think we weren’t just connecting with the right people, but we have since been able to work with some of our local communities, and we are involved in some projects that are supporting adaptation planning within First Nations communities within our region, and so, that was really great, having some conversations with an individual at First Nations who was doing some climate change adaptation, and I was wanting to see what they were doing and then meeting her at other conferences, and that allowed us to build a relationship, and she then brought us into her projects that she’s doing. So that has been really great because we saw a gap in our vulnerability assessment …we didn’t have any Indigenous views throughout the vulnerability assessment…. and so, now that we have those connections, it has been great, and there has been a real collaborative approach to being able to support them when we can with our vulnerability assessment and they are using traditional ecological knowledge as well to further adaptation planning, so it has been really great to be able to start to build those relationships.” [PH participant]	Local
**Advanced coal phase-out**	“ [Redacted] appeared before the legislative committee and conservatives said, this is a waste of time, we don’t need a legislation, we have already stopped burning coal for electricity…We said that we want to be there a legislation so that anybody who tries to reverse it, it’s going to be harder to do that. And everyone was kind of like, we will never go back, and then what happened two years later? You know, you had a president [referring to U.S politics] who was now calling for burning of coal. So, I would say that was our role. So I’ll just say, we have had this tradition of speaking out about the links between the environment and health and we are going to continue to do that.” [PH participant]	Provincial
